# Protection against Experimental Melioidosis following Immunisation with a Lipopolysaccharide-Protein Conjugate

**DOI:** 10.1155/2014/392170

**Published:** 2014-05-07

**Authors:** Andrew E. Scott, Sarah A. Ngugi, Thomas R. Laws, David Corser, Claire L. Lonsdale, Riccardo V. D'Elia, Richard W. Titball, E. Diane Williamson, Timothy P. Atkins, Joann L. Prior

**Affiliations:** ^1^Defence Science and Technology Laboratory, Porton Down, Salisbury SP4 0JQ, UK; ^2^Fleet Bioprocessing Ltd., Pale Lane Farm, Hartley Wintney RG27 8DH, UK; ^3^College of Life and Environmental Sciences, University of Exeter, Exeter EX4 4QD, UK

## Abstract

Melioidosis is a severe infectious disease caused by *Burkholderia pseudomallei*. It is refractory to antibiotic treatment and there is currently no licensed vaccine. In this report we detail the construction and protective efficacy of a polysaccharide-protein conjugate composed of *B. pseudomallei* lipopolysaccharide and the H_c_ fragment of tetanus toxin. Immunisation of mice with the lipopolysaccharide-conjugate led to significantly reduced bacterial burdens in the spleen 48 hours after challenge and afforded significant protection against a lethal challenge with *B. pseudomallei*. The conjugate generated significantly higher levels of antigen-specific IgG1 and IgG2a than in lipopolysaccharide-immunised mice. Immunisation with the conjugate also demonstrated a bias towards Th1 type responses, evidenced by high levels of IgG2a. In contrast, immunisation with unconjugated lipopolysaccharide evoked almost no IgG2a demonstrating a bias towards Th2 type responses. This study demonstrates the effectiveness of this approach in the development of an efficacious and protective vaccine against melioidosis.

## 1. Introduction

*Burkholderia pseudomallei* is a Gram-negative soil-dwelling bacterium which is readily recovered from the environment in Southeast Asia and Northern Australia [1, 2]. It is the etiological agent of melioidosis, a severe disease in which infection is thought to be due to inhalation of water or soil particles or contamination of skin abrasions [3]. Acute septicaemic melioidosis is responsible for much morbidity and mortality, especially in north-eastern Thailand where melioidosis is the most common cause of community-acquired septicaemia [4, 5]. The disease may also manifest as an undetected or subclinical infection, persisting for a number of years until activated by a traumatic event or a decrease in immunocompetence [6].* B. pseudomallei *is highly virulent via the aerosol route in a number of animal models [7–9], and because of these factors melioidosis is today regarded as an emerging infectious disease and is listed as a CDC tier 1 Select Agent.* B. pseudomallei *is highly refractory to antibiotic treatment [10], suggesting that vaccination might be the most effective way of controlling melioidosis [11]. Significant effort has been directed towards identifying and testing candidate vaccines, but thus far there are no candidates which are nearing licensure (reviewed by [12–14]).

A range of surface polysaccharides have been identified in* B. pseudomallei*, and lipopolysaccharide (LPS) and capsular polysaccharide have been evaluated as vaccine candidates [15]. The O-polysaccharide of the lipopolysaccharide (LPS) is an unbranched heteropolymer consisting of variably acetylated and methylated -3)-*β*-D-glucopyranose-(1-3)-6-deoxy-*α*-L-talopyranose-(1- [16–18] and has been reported to play a role in resistance to serum killing and virulence [19]. Monoclonal and polyclonal antibodies raised against LPS passively protect against* B. pseudomallei* infection in a number of animal models [15, 20–22]. However, immunisation with purified LPS from* B. pseudomallei *provided only 50% protection in a mouse model of melioidosis and did not result in clearance of infection in the survivors [15]. The O-antigen of* B. pseudomallei *has previously been used in a conjugate vaccine and the sera generated from these vaccinations provided passive protection in diabetic rats [21], although protection using an active immunisation strategy was not examined.

A number of polysaccharide vaccines are currently licensed including those to combat* Haemophilus influenzae*,* Neisseria meningitidis*, and* Streptococcus pneumoniae* infection. Polysaccharides generally elicit a T-independent immune response with the production of IgM and IgG3 antibodies and a general failure to switch to IgG production [23]. In order to convert the response to a more favourable T-dependent response and to induce T-cell memory, polysaccharides can be conjugated to proteins [23, 24]. This is the case with the* H. influenzae* type b vaccine and the* meningococcal* type C vaccines which are licensed for clinical use. In this report we detail the construction and use of an LPS-protein conjugate vaccine which generates balanced immune responses and provides effective protection against melioidosis in a murine model of infection.

## 2. Methods and Materials

### 2.1. LPS Purification and Analysis

The nonencapsulated* B. pseudomallei *K96243 Δ*wcbH *mutant [25] was used to prepare capsule-free LPS. This strain was grown for48 hours on L-agar and the bacterial growth harvested into PBS using sterilised glass beads. Following heat-killing and subsequent lyophilisation, LPS was recovered using a previously described hot-phenol extraction method [15, 26]. The recovered LPS was resuspended in sterile distilled water and its purity was assessed using SDS polyacrylamide gel electrophoresis (PAGE) and western blotting. Tris-glycine SDS PAGE was performed using a 12.5% separating gel with a 4.5% stacking gel in a Mini-PROTEAN tetra cell system (Bio-Rad). Gels were silver-stained according to the method of chart [27]. To assess the antigenicity of the purified LPS and confirm the absence of capsule polysaccharide, samples were separated by SDS PAGE and blotted to nitrocellulose membranes with a semidry transfer system. These membranes were probed using anti-LPS or anti-CPS antibodies as the primary [20] and anti-mouse horseradish peroxidase labelled conjugate as the secondary antibody (Sigma-Aldrich). The membranes were developed with SigmaFAST DAB (Sigma-Aldrich).

### 2.2. TetH_c_ Production

Recombinant tetanus toxin H_c_ fragment (TetH_c_, incorporating amino acids 865–1315 from TetX (NP_783831) of* Clostridium tetani* E88) was recovered from* E. coli* BL21 (pKS1-TetH_c_), kindly supplied by Dr. N. Fairweather [28]. Briefly, cells were grown, induced, and harvested using the method of Sinha et al. [28] and the recombinant His-tagged protein recovered using HisTrap HP columns (GE Healthcare) on an Akta FPLC with elution in steps up to 500 mM imidazole. Following dialysis, the recovered protein was assessed for purity using Coomassie stained SDS PAGE gels and the concentration determined using a BCA assay (Pierce).

### 2.3. Conjugation

LPS and TetH_c_ were conjugated via the short chain heterobifunctional spacer reagents N-succinimidyl S-acetylthioacetate (SATA; Pierce) and 3,3′-N-[*ε*-Maleimidocaproic acid] hydrazide (EMCH; Pierce). Briefly, TetH_c_ was derivatised with SATA following buffer exchange into 0.1 M potassium phosphate (pH 7.5) using PD10 columns (GE Healthcare). The concentration of TetH_c_ was determined after desalting using A_280_ readings. SATA was made up to 10 mg/mL in DMSO and immediately added to a final concentration of 12 molar equivalents of SATA to TetH_c_. After incubating at 20°C for 60 minutes, the reaction was quenched with 10% hydroxylamine for 15 minutes and the resulting thiolated protein was desalted into conjugation buffer (50 mM potassium phosphate, 150 mM NaCl, 5 mM EDTA, pH 7.0) and quantified using A_280_ readings. Thiol incorporation was determined using Ellman's reagent (Pierce). The thiolated TetH_c_ protein was reacted with the derivatised LPS prepared as below within 10 minutes of the final desalting step.

LPS was reconstituted with 50 mM 2-(N-morpholino)ethanesulfonic (MES) acid buffer (pH 5.5) for derivitisation with EMCH. To this solution were added 100 molar equivalents of N-hydroxysuccinimide (NHS) in MES buffer and 100 molar equivalents of 1-Ethyl-3-[3-dimethylaminopropyl] carbodiimide hydrochloride (EDC) in MES buffer. The resulting solution was mixed at 20°C for 15 minutes and then 50 molar equivalents of EMCH in DMSO were added. After mixing for 15 minutes, the pH was adjusted to 7.0 and the resulting solution was stirred for further 60 minutes. The activated LPS was then desalted into PBS + 5 mM EDTA and reacted with the derivatised TetH_c_ as below.

The two derivatised solutions were mixed and incubated at 20°C for two hours. After quenching with 2-mercaptoethanol and N-ethylmaleimide, the solution was filtered, concentrated, and purified into presentation buffer (50 mM potassium phosphate, 150 mM NaCl, pH 6.7) on a 2.6 × 25 cm Superdex 200 PG column. The recovered fractions were concentrated and filtered for assessment.

### 2.4. Conjugate Analysis

The conjugate was analysed using a variety of methods to assess the relative concentrations of protein and LPS. Protein concentration was determined using a BCA protein assay. The concentration of LPS was measured using a phenol sulphuric acid assay [29] with comparison to a known concentration of purified LPS using glucose to generate a standard curve. The conjugate was assessed for relative size compared to unconjugated TetH_c_, both by its elution time off the Superdex column after conjugation and by visually using Coomassie staining following separation on Novex tris-acetate native gels (Invitrogen). A capture enzyme-linked immunosorbent assay (ELISA) was also used to analyse the conjugate and to confirm the covalent linkage between the LPS and TetH_c_. This was done as described previously [30] with minor modifications. Briefly, 96-well plates were coated with 10 *μ*g/mL of mouse-derived anti-LPS monoclonal antibody in PBS. Sample was added at a set starting concentration (1 : 5) and double diluted down the plate. This was probed using 10 *μ*g/mL rabbit-derived anti-TetH_c_ antibody with subsequent detection by horseradish peroxidase (HRP) labelled anti-rabbit antibody (Sigma-Aldrich) used at 1 : 5000. The plates were developed and read using ABTS (Sigma-Aldrich).

### 2.5. Animal Studies

Studies were performed using 6- to 8-week-old female BALB/c mice (Charles River). Animals were randomly grouped together on arrival in cages of five or six mice with* ad libitum* access to food and water under a 12-hour light/dark cycle. After challenging with viable* B. pseudomallei*, the animals were handled under ACDP containment level 3 conditions within a half-suit isolator, compliant with British Standard BS5726. All investigations involving animals were carried out according to the requirements of the Animal (Scientific Procedures) Act 1986.

Studies were performed on two independent occasions. Each study was composed of two parts: postchallenge monitoring using groups of 5 mice in each study and vaccine efficacy using groups of 6 mice and 10 mice in the first and second studies, respectively. Mice were immunised three times (days 0, 14, and 28) with 0.1 mL of vaccine via the intraperitoneal route. Each dose of conjugate contained the equivalent of 10 *μ*g of LPS, whilst the control groups received matching amounts of free LPS, free LPS mixed with TetH_c_, TetH_c_ only, or PBS. No adjuvant was used in any of the vaccine groups. Four weeks after the final boost, mice were tail-bled to recover serum for analysis. A further week later, the mice in the two studies were challenged via the intraperitoneal route with 4.0 × 10^4^ cfu and 4.2 × 10^4^ cfu (approximately 40 MLD) of* B. pseudomallei *K96243, respectively. Mice in the postchallenge monitoring groups were culled 48 hours after challenge and spleens were removed for bacteriological and immunological assessment. Mice in the efficacy groups were monitored twice daily up to day 29 after challenge, when the surviving mice were culled and spleens were removed for bacteriological assessment.

### 2.6. Analysis of Antibody Responses

Approximately 100 *μ*L of blood was removed from the tail vein of all mice one week prior to challenge with* B. pseudomallei*. The blood was allowed to clot at 4°C for 24 hours and centrifuged at 13,000 ×g and the serum was removed and stored at −20°C. Subsequently, the responses directed against* B. pseudomallei *LPS were assessed by an ELISA as previously described [30] with minor modifications. Briefly, 96-well plates were coated with 5 *μ*g/mL of LPS purified from* B. pseudomallei *K96243 Δ*wcbH*. The primary antibody was the relevant mouse serum added at a set starting concentration (typically 1 : 50) and double diluted down the plate. The secondary antibody was the relevant HRP labelled anti-mouse isotype antibody (Sigma-Aldrich) used at 1 : 5000. The plates were developed and read using ABTS. Each 96-well plate contained a standard curve of wells coated overnight with 5 *μ*g/mL anti-mouse FAB in PBS then loaded with a dilution of the relevant mouse isotype control (Sigma-Aldrich) starting at 0.2 *μ*g/mL. The secondary antibody was the relevant anti-mouse HRP conjugate. The concentration of LPS-specific antibodies of each sample was calculated by comparison to the standard curve using sera recovered from PBS immunised mice as the negative control.

### 2.7. Postinfection Analysis

Groups of mice were culled two days after challenge and their spleens were removed into PBS. The spleens were then mashed through 70 *μ*m cell sieves to create a cell suspension. Bacterial load within the spleen was determined by serial dilution. Of the remaining cell suspension, 200 *μ*L aliquots were taken and centrifuged at 2000 ×g for 5 minutes. The supernatants were recovered and stored at −80°C. Mouse inflammatory cytometric bead arrays (BD biosciences) were performed on the supernatants in accordance with manufacturer's instructions and measured using a BD FACS CANTO flow cytometer.

## 3. Results

### 3.1. Conjugate Synthesis

The strategy chosen to generate the LPS-TetH_c_ conjugate used in this study resulted in the covalent joining of LPS and TetH_c_ via a thioether bond between primary amine groups in the TetH_c_ and carboxyl groups in the LPS. The heterobifunctional short-chain linker molecules SATA and EMCH were used to effect this bond formation through the introduction of reactive sulfhydryl and maleimide groups onto the amine and carboxyl groups, respectively. This follows on from a previous study looking at a number of LPS-protein conjugates which determined that the inclusion of a spacer molecule led to increased immunogenicity [21]. This reaction scheme used a total of 21 mg of LPS and 8.1 mg of TetH_c_. Derivitisation of 8.1 mg of TetH_c_ using SATA yielded a total of 7.5 mg of derivatised TetH_c_ at 0.54 mg/mL with a calculated 3.8 thiols per TetH_c_ molecule. A total of 21 mg of LPS at 5 mg/mL initial concentration were used for derivitisation with EMCH. A total of 29 mL of LPS-TetH_c_ were recovered after conjugation and this was determined to have a protein concentration of 0.35 mg/mL and a glycan content equivalent to a 1 mg/mL solution of purified LPS. Although LPS is a heterogeneous molecule with its molecular weight ranging from 20 to 80 kDa, if a figure corresponding to the most commonly observed size by SDS-PAGE [13] of 45 kDa is used for calculations, this gives 3.2 molecules of LPS per molecule of TetH_c_. This corresponds well with the 3.8 thiols per TetH_c_ observed after TetH_c_ derivitisation and indicates efficient conjugation between the LPS and TetH_c_.

**Figure 1:**
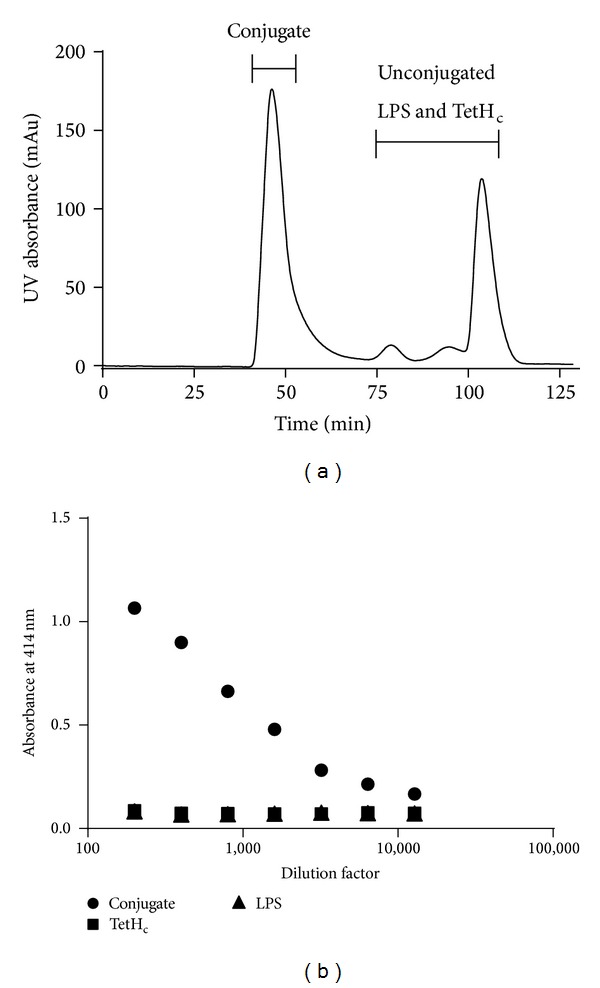
Conjugate analysis. FPLC trace detailing elution of the TetH_c_-LPS conjugate following synthesis (a). The recovered conjugate fraction (*∙*) was analysed by capture ELISA (b) with comparison to unconjugated LPS (▲) and unconjugated TetH_c_ (■). Only the conjugate, which contains both antigens in a single molecule, provided a positive result.

To demonstrate the presence of a covalent linkage between the LPS and TetH_c_ molecules after conjugation, a capture ELISA was performed using both anti-LPS and anti-TetH_c_ antibodies. The conjugate, having both antigens in a single molecule, had absorbance readings which were higher than background when used at dilutions up to 1 : 12,800. By contrast, equivalent amounts of free LPS and free TetH_c_ molecules were negative even at a 1 : 200 dilution (Figure 1). Subsequent examination of the conjugate by acrylamide gel electrophoresis similarly demonstrated that the conjugate was composed of molecules with an apparent molecular weight in excess of 100 kDa, whereas the individual LPS and TetH_c_ were smaller molecules. Although it was not possible to assign a formal structure to the conjugate, the conjugate was able to enter and migrate in gels under nonreducing conditions, something that would be unlikely for cross-linked conjugates in a lattice formation and suggests that the conjugate consists of discrete molecules of linked TetH_c_ and LPS.

### 3.2. Immunisation with Conjugated LPS Provides Protection against Experimental Melioidosis

**Figure 2:**
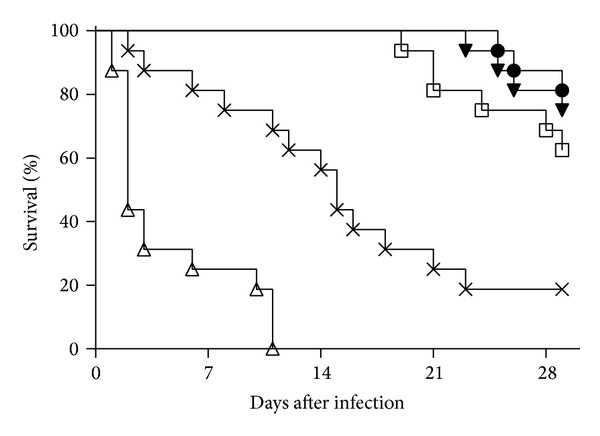
Survival of vaccinated mice following challenge with* B. pseudomallei* K96423. Mice were vaccinated three times at two-week intervals with 10 *μ*g of either the TetH_c_-LPS conjugate (*∙*), LPS alone (□), a mix of unconjugated LPS and TetH_c_ (*▾*), TetH_c_ only (Δ), or PBS (**x**) and challenged five weeks after the final vaccination with approximately 40 MLD of* B. pseudomallei *K96243. The data from two studies is combined in this figure with survival curves analysed by a stratified logrank test. We found no evidence (*P* > 0.05) for cross experimental variation in any vaccine group other than the TetH_c_ group where animals survived less well in the second experiment (*P* < 0.05). All of the vaccine containing* B. pseudomallei* LPS offered significant protection compared to the unvaccinated control (*P* < 0.001, in all cases) and the TetH_c_ treated group (*P* < 0.001, in all cases). We observed no statistical difference between the groups vaccinated with conjugate, LPS, or the mix of TetH_c_ and LPS (*P* > 0.05, in all cases).

Groups of mice were immunised as described, challenged with* B. pseudomallei *K96243, and monitored over the subsequent 29 days. Whereas all but three of the PBS immunised mice succumbed to infection by the end of the study, 81% of the mice immunised with the LPS-TetH_c_ conjugate survived to day 29 after challenge. Immunisation with free LPS only or a mix of free LPS and TetH_c_ resulted in 62% and 75% survival, respectively (Figure 2). Thus, immunisation with any form of LPS provided significant protection against experimental melioidosis compared to the groups receiving a PBS vaccine (*P* < 0.001 in all cases). Conversely, survival in groups immunised with the TetH_c_ protein only was significantly lower than in the PBS immunised groups (*P* < 0.01), with the mice rapidly succumbing to infection.

At 29 days after challenge, spleens were removed from the surviving mice and examined for bacterial burden. The majority of mice in all groups were harbouring* B. pseudomallei *in their spleens, with numbers ranging between 1 × 10^2^ cfu and 1 × 10^7^ cfu per spleen, although a limited number had cleared the infection at this point. There were no statistically significant differences in the splenic burdens of the mice in the different vaccine groups, or in the numbers of mice in each vaccine group which had cleared the infection (data not shown).

### 3.3. Conjugated LPS Generates More Diverse Antibody Responses

**Figure 3:**
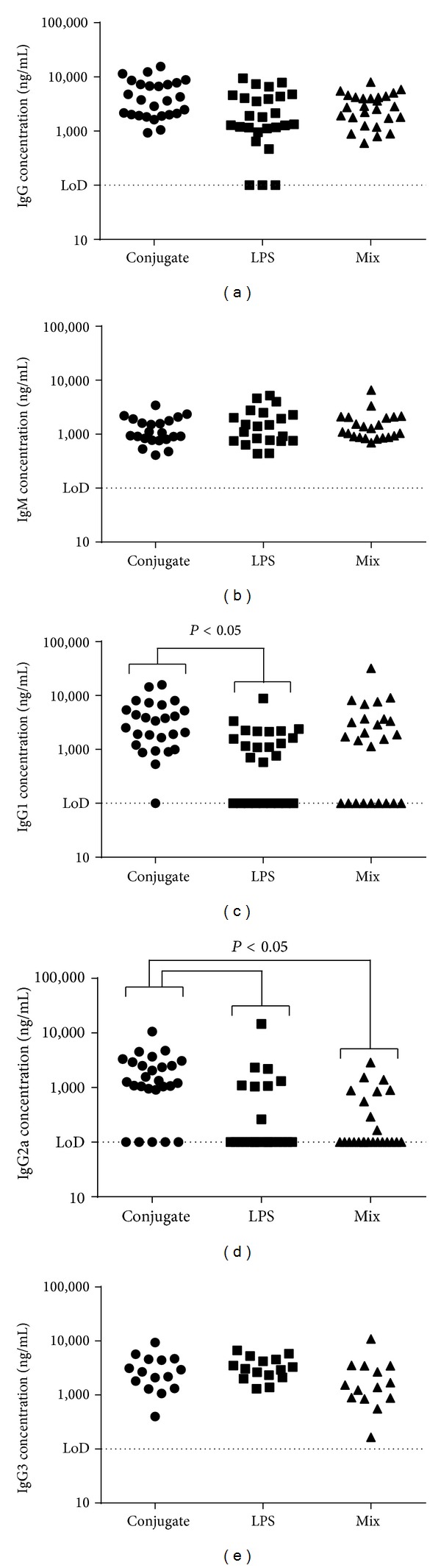
Antibody concentrations in serum following vaccinations. Animals received three vaccinations at two-week intervals each of 10 *μ*g of either the TetH_c_-LPS conjugate (*∙*), LPS alone (■), or a mix of unconjugated LPS and TetH_c_ (▲). Tail bleeds were performed four weeks after the final vaccination and ELISA performed to detect antibodies specific for* B. pseudomallei *LPS. Data is shown for replicate studies and shows the calculated concentration of total IgG (a), total IgM (b), IgG1 (c), IgG2a (d), and IgG3 (e). Data was analysed by a univariate linear model. Significant differences are shown. Where no markers are present, *P* was greater than 0.05.

A potential advantage of conjugate polysaccharide vaccines is the elicitation of T-cell help for B-cells producing specific IgG antibodies, leading to class switching from IgM to IgG and a more sustained memory response [23, 24]. To assess the immunogenicity of the various vaccines in these studies, all immunised mice were tail-bled four weeks after the final boost and levels of serum antibodies were assessed using an ELISA (Figures 3(a) and 3(b)). All groups produced roughly equivalent levels of IgM and IgG which were not statistically different (*P* > 0.05). The polarisation of the immune response was assessed (Figures 3(c) and 3(d)) through analysis of levels of LPS-specific IgG1, IgG2a, and IgG3, the relative proportions of which are held to reflect the bias of an immune response in the mouse [31]. A significant number of the mice immunised with LPS alone failed to produce any detectable IgG1 or IgG2a (26% had no detectable IgG1 and 69% had no detectable IgG2a). This was also true for mice immunised with a mix of LPS and TetH_c_ (36% had no detectable IgG1 and 64% had no detectable IgG2a), whereas mice receiving the conjugate generally produced strong levels of both IgG1 and IgG2a (4% failed to produce detectable levels of IgG1 and 19% had no detectable IgG2a). In this respect, the differences between the conjugate group and the LPS only and mix of LPS and TetH_c_ groups were significant (*P* < 0.02 for IgG1 and *P* < 0.0001 for IgG2a by Fisher's Exact Tests). Taking this into account, mice receiving the conjugate had significantly higher titres of both IgG1 and IgG2a compared to mice immunised with LPS alone (*P* < 0.05) and significantly higher titres of IgG2a compared to mice receiving a mix of unconjugated LPS and TetH_c_ (*P* < 0.05), indicating that immunisation with the conjugated LPS generated a more diverse range of immune responses.

### 3.4. Immunisation Provides Early Responses

**Figure 4:**
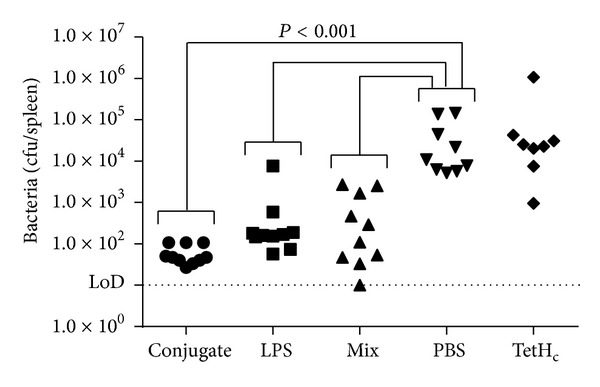
The bacterial burdens of spleens 48 hours after infection. Mice were vaccinated three times at two-week intervals with 10 *μ*g of either the TetH_c_-LPS conjugate (*∙*), LPS alone (■), a mix of unconjugated LPS and TetH_c_ (▲), TetH_c_ only (*◆*), or PBS (*▾*) and challenged five weeks after the final vaccination with approximately 40 MLD of* B. pseudomallei *K96243. Forty-eight hours after challenge, mice were culled and spleens were removed and assayed for bacterial burden. Data is shown for replicate studies and was analysed by univariate linear model. Significant differences are shown. Where no markers are present, *P* was greater than 0.05.

In order to assess the impact of the different vaccines in the early stages of infection, immunised mice were culled 48 hours after* B. pseudomallei *challenge and bacterial burdens and levels of proinflammatory cytokines in the spleen measured. All of the mice were found to be carrying* B. pseudomallei *in their spleens (Figure 4). However, those mice which received a vaccine composed of any form of LPS resulted in an approximately 100-fold reduction in bacterial burden in the spleen compared to the PBS immunised mice. These reductions were statistically significant in all cases (*P* < 0.001). There were no significant differences in the spleen counts between the mice receiving conjugate vaccine as opposed to the mice receiving nonconjugated vaccines, although in both experiments the mean bacterial burden was between 2-fold and 5-fold lower in the conjugate vaccine group when compared to the LPS only immunised group. The control mice receiving TetH_c_ only as a vaccine had similar splenic burdens as the PBS immunised mice.

**Figure 5:**
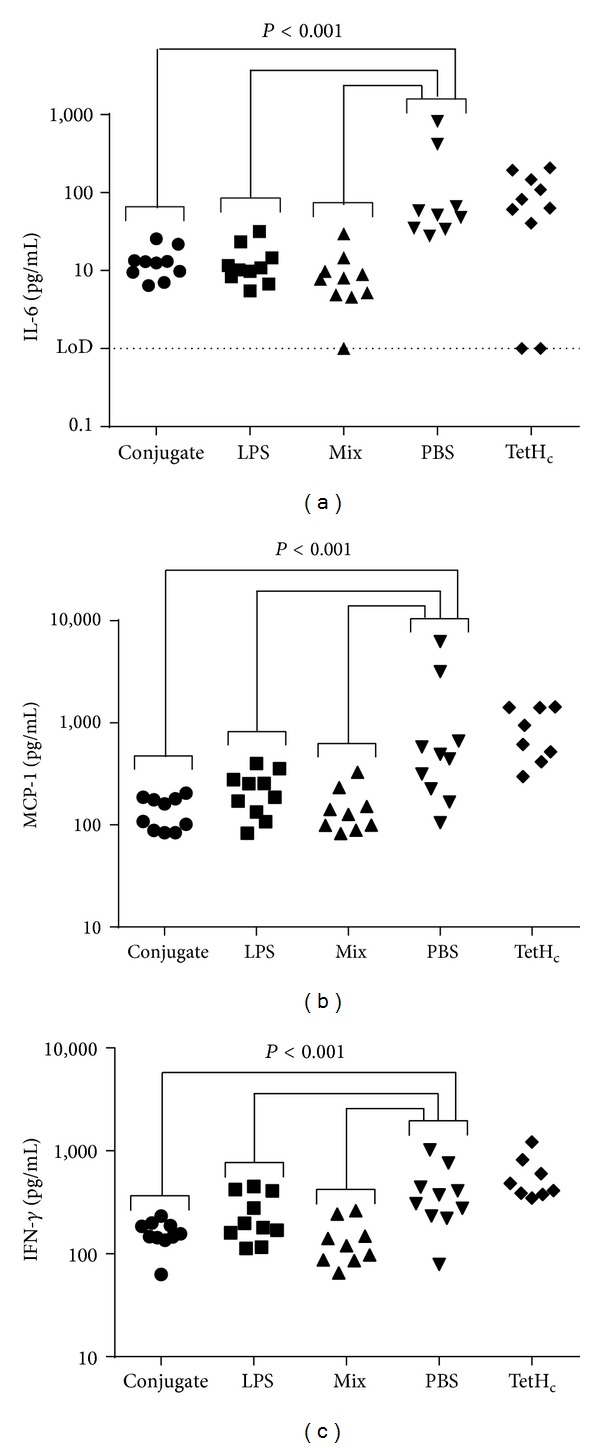
Cytokine production in spleens 48 hours after infection. Mice were vaccinated three times at two-week intervals with 10 *μ*g of either the TetH_c_-LPS conjugate (*∙*), LPS alone (■), a mix of unconjugated LPS and TetH_c_ (▲), TetH_c_ only (*◆*), or PBS (*▾*) and challenged five weeks after the final vaccination with approximately 40 MLD of* B. pseudomallei *K96243. Forty-eight hours after challenge, mice were culled and the spleens were removed and assayed for cytokine production. Data is shown for replicate studies and shows the calculated concentration of IL-6 (a), MCP-1 (b), and IFN-*γ* (c) and was analysed by univariate linear model. Significant differences are shown. Where no markers are present, P was greater than 0.05.

The levels of key proinflammatory cytokines were measured in spleens using cytometric bead arrays (Figure 5). Cytokines are released in response to the presence of bacterial antigen and therefore their levels will correlate well with infectious load. Further, any deviation from this correlation between infectious load and cytokine will be likely due to the influence of the vaccines. The mice receiving a PBS vaccine had significantly higher levels of IL-6, MCP-1, and IFN-*γ* compared to those receiving any form of LPS (*P* < 0.05 in all cases). Contrastingly, mice receiving TetH_c_ only as a vaccine had significantly higher levels of proinflammatory cytokine than even the PBS immunised mice (*P* < 0.001). These mice, however, did not have significantly increased bacterial loads when compared to their PBS immunised counterparts, suggesting that despite producing more cytokine this was not effectively controlling the infection.

## 4. Discussion

The aim of this study was to produce and characterise an LPS-based conjugate vaccine against experimentally induced melioidosis. Our work included characterisation of the immune response elicited by the conjugate and determination of its protective efficacy. In designing the conjugation chemistry to be used, there was some concern that immunologically relevant epitopes in the LPS would be lost during the conjugation process. In particular, it is known that* O*-acetylation of the deoxy-talose is important for differential reactivity towards monoclonal antibodies between the LPSs of* B. mallei *and* B. pseudomallei *[32]. The generation of a protective immune response in this study clearly indicates that the conjugate vaccine retains the protective epitopes found in LPS, despite being exposed to a number of reactive chemicals during the conjugation procedure, and that this conjugation strategy is of use for the development of future LPS-based glycoconjugate vaccines.

One of the potential advantages of polysaccharide conjugate vaccines is the elicitation of T-cell help for B-cells producing IgG antibodies, leading to class switching from IgM to IgG and the establishment of a memory response [23, 24]. Examination of prechallenge sera indicated that all of the immunised mice generated broadly equivalent levels of IgM and IgG. This likely reflects that* B. pseudomallei *LPS has been reported to stimulate several Toll-like receptors [33] and as such is capable of stimulating the immune system in a way which effectively drives the development of B-cells to generate higher levels of IgG than would be expected from this T-independent antigen [34]. However, although total IgG levels in the different vaccine groups were equivalent, more detailed examination of the different subtypes of IgG produced indicated that the conjugate vaccine was uniquely able to drive the development of a more diverse immune response. This was characterised by elevated levels of IgG1 and IgG2a, suggestive of a T-dependent response mediated by covalent attachment to TetH_c_. In contrast, in the majority of mice immunised with unconjugated LPS, the bias was towards production of IgG3 antibodies with little or no IgG1 or IgG2a, suggestive of a T-independent response [23].

As expected, immunisation with the conjugate provided significant protection compared with control mice receiving PBS (*P* < 0.0001), with 81% of mice surviving to the end of the study. This was also true for vaccines containing unconjugated LPS, although survival was lower in these cases (62% and 75% for LPS only and the LPS/TetH_c_ mix, respectively). Previous studies have shown that immunisation with* B. pseudomallei *or* B. thailandensis *LPS can protect roughly half of immunised mice [15, 26], and the results of this study are broadly in line with those findings. As a polysaccharide LPS is unlikely to stimulate much cell-mediated immunity, so protection is likely to be mediated by antibodies. That the conjugate generated a range of antibodies which are characteristic of a more developed humoral response, and yet the conjugate-immunised mice were not protected to a significantly greater degree than those receiving unconjugated LPS, is disappointing. TetH_c_ is unlikely to contribute any T-cell help specific for* Burkholderia*. Therefore, the basis of protection is LPS-directed antibody. Even though conjugation to protein appears to act to increase the range of isotypes of LPS-directed antibody, this has not given a step change in protection. It is possible that this reflects that complete protection by subunit vaccines cannot rely on antibodies alone; unconjugated LPS generated sufficient antibodies to provide the protection observed, but going beyond this level of protection cannot be achieved without engaging other facets of the immune response, particularly a* Burkholderia*-specific cell-mediated response [35].

None of the vaccines described in this study allowed all mice to clear the infection, something which is desirable given the potential for* B. pseudomallei *to persist in the host and to cause chronic disease. Although immunisation did not lead to clearance, it did lead to a greatly extended time to death, with mice receiving the conjugate surviving for longer than the mice receiving LPS alone. This extended time to death translates to an increased window of opportunity for antibiotic intervention, a very positive factor considering the generally acute nature of community-acquired melioidosis, manifesting in 50% mortality within 48 hours of arrival at hospital, and the increasingly likely scenario that the combined use of immunisation and antibiotic therapy may be the most appropriate choice for treatment of melioidosis [13]. However, further work would be needed to assess the impact of immunisation on the progression of clinical symptoms, diagnosis, and triggers to treat with antibiotics.

It is known that the cytokine interferon-*γ* (IFN-*γ*) is of critical importance for the control of melioidosis, since IFN-*γ* knockout mice succumbed to infection much more rapidly than immunocompetent mice [36, 37]. However, it is also clear that the level of IFN-*γ* does not necessarily correlate directly with ability to clear infection; susceptible mice such as BALB/c display highly elevated levels of IFN-*γ* compared to innately resistant mice such as C57Bl/6 mice [38, 39]. Our results indicate that IFN-*γ* levels 48 hours after challenge showed an inverse correlation with the outcome of infection; PBS-immunised mice showed the highest levels of IFN-*γ* but the poorest outcome whilst mice immunised with the conjugate showed the lowest IFN-*γ* levels and the best outcome. Our results lend weight to the possibility that it is the temporal pattern of IFN-*γ* production which is important for control of infection. In essence, naïve mice display a “too much too late” phenotype as proposed to occur in pneumonic tularaemia [40]. Further work is required to investigate this possibility.

Unexpectedly, mice vaccinated with the TetH_c_ fragment succumbed to the disease more rapidly than their PBS vaccinated counterparts (*P* < 0.001) with their median time to death lowered from 14 days to 2 days. This was observed in two separate studies performed eight months apart. This exacerbation of disease was entirely tempered by the addition of LPS. The tetanus toxin from which TetH_c_ was derived is not a* B. pseudomallei *protein, and detailed searches through DNA and protein sequences coupled with immunoscreening using anti-TetH_c_ antibodies failed to identify any homologous proteins in* B. pseudomallei*. At present, the cause of this phenomenon remains to be determined. It should be made clear that this work did not use the licensed tetanus vaccine, which is the toxoided full length toxin, and the data should not be extrapolated to argue that this vaccine should not be taken in areas where melioidosis is endemic. The tetanus vaccine is fully licensed following clinical trials and has been shown to be safe and efficacious against a potentially fatal disease. Additionally, several studies have looked at risk factors for melioidosis, and immunisation against tetanus has not been identified [41–44]. We are currently investigating this interesting observation.

In this paper, we have demonstrated that the conjugation of LPS to a carrier protein generates a vaccine which elicits broad activation of the immune system. The conjugate vaccine offered significant protection against experimental melioidosis. Further work is still needed to develop this as a candidate appropriate for licensure; in particular, there is the need to remove the endotoxic lipid A and to add a suitable adjuvant, but this paper demonstrates the effectiveness of this conjugation strategy in the development of an efficacious vaccine against melioidosis.
